# Versatile Loading of Diverse Cargo into Functional Polymer Capsules

**DOI:** 10.1002/advs.201400007

**Published:** 2015-01-29

**Authors:** Joseph J. Richardson, James W. Maina, Hirotaka Ejima, Ming Hu, Junling Guo, Mei Y. Choy, Sylvia T. Gunawan, Lien Lybaert, Christoph E. Hagemeyer, Bruno G. De Geest, Frank Caruso

**Affiliations:** ^1^ ARC Centre of Excellence in Convergent Bio‐Nano Science and Technology and the Department of Chemical and Biomolecular EngineeringThe University of MelbourneParkvilleVictoria3010Australia; ^2^ Vascular Biotechnology LaboratoryBaker IDI Heart and Diabetes InstituteMelbourneAustralia; ^3^ Department of PharmaceuticsGhent UniversityGhentBelgium

**Keywords:** drug delivery, drug loading, inorganic templates, nanomedicine, polymer capsules

## Abstract

Polymer microcapsules are of particular interest for applications including self‐healing coatings, catalysis, bioreactions, sensing, and drug delivery. The primary way that polymer capsules can exhibit functionality relevant to these diverse fields is through the incorporation of functional cargo in the capsule cavity or wall. Diverse functional and therapeutic cargo can be loaded into polymer capsules with ease using polymer‐stabilized calcium carbonate (CaCO_3_) particles. A variety of examples are demonstrated, including 15 types of cargo, yielding a toolbox with effectively 500+ variations. This process uses no harsh reagents and can take less than 30 min to prepare, load, coat, and form the hollow capsules. For these reasons, it is expected that the technique will play a crucial role across scientific studies in numerous fields.

## 1 Introduction

Polymer microcapsules are of particular interest for applications including self‐healing coatings,^1^ catalysis,^2^, ^3^ bioreactions,^4^, ^5^ sensing,^3^, ^6^ and drug delivery.^7^, ^8^ The primary way that polymer capsules can exhibit functionality relevant to these diverse fields is through the incorporation of functional cargo in the capsule cavity or wall. Detailed loading methods exist but are mainly dependent on the properties of the cargo. For example, the solubility,^9^ charge,^5^ radius of gyration or size,^10^ and chemical composition^8^, ^11^ of the cargo often determine how it can be loaded into polymer capsules. A particular challenge is the incorporation of small molecules, as water soluble drugs have a tendency to “leak” out of the capsules,^12^ due to the semipermeable nature of many polymer films.^13^, ^14^ A related challenge is the incorporation of large materials^15^ (on the order of tens to hundreds of nanometers) that are thicker than most polymer capsule walls^16^ (on the order of nanometers) and are therefore difficult to incorporate or embed in the coatings. The use of calcium carbonate (CaCO_3_) particles for the preparation of loaded polymer capsules has been well studied, especially because CaCO_3_ particles can be coprecipitated with proteins, are highly biologically compatible, and can be made in unique geometries.^17^, ^18^ However, coprecipitated CaCO_3_ particles and the resultant polymer capsules are generally polydisperse and require the addition of a stabilizing polymer (e.g., poly(styrene sulfonate), PSS) during formation to allow for monodisperse spherical CaCO_3_ particles (PSS–CaCO_3_ particles) to be prepared.^19^ In previous reports, small molecules were loaded into these particles and polymer capsules could be subsequently prepared following layer‐by‐layer (LbL) coating with multiple polymer layers.^17^, ^19^, ^20^, ^21^ However, the capacity of PSS–CaCO_3_ particles to adsorb high amounts of diverse cargo ranging in size, charge, and hydrophobicity has not been explored, nor has it been reported that capping with a single polymer layer, rather than capping with a LbL film,^17^, ^19^, ^20^, ^21^ is sufficient for capsule preparation.

**Scheme 1:**
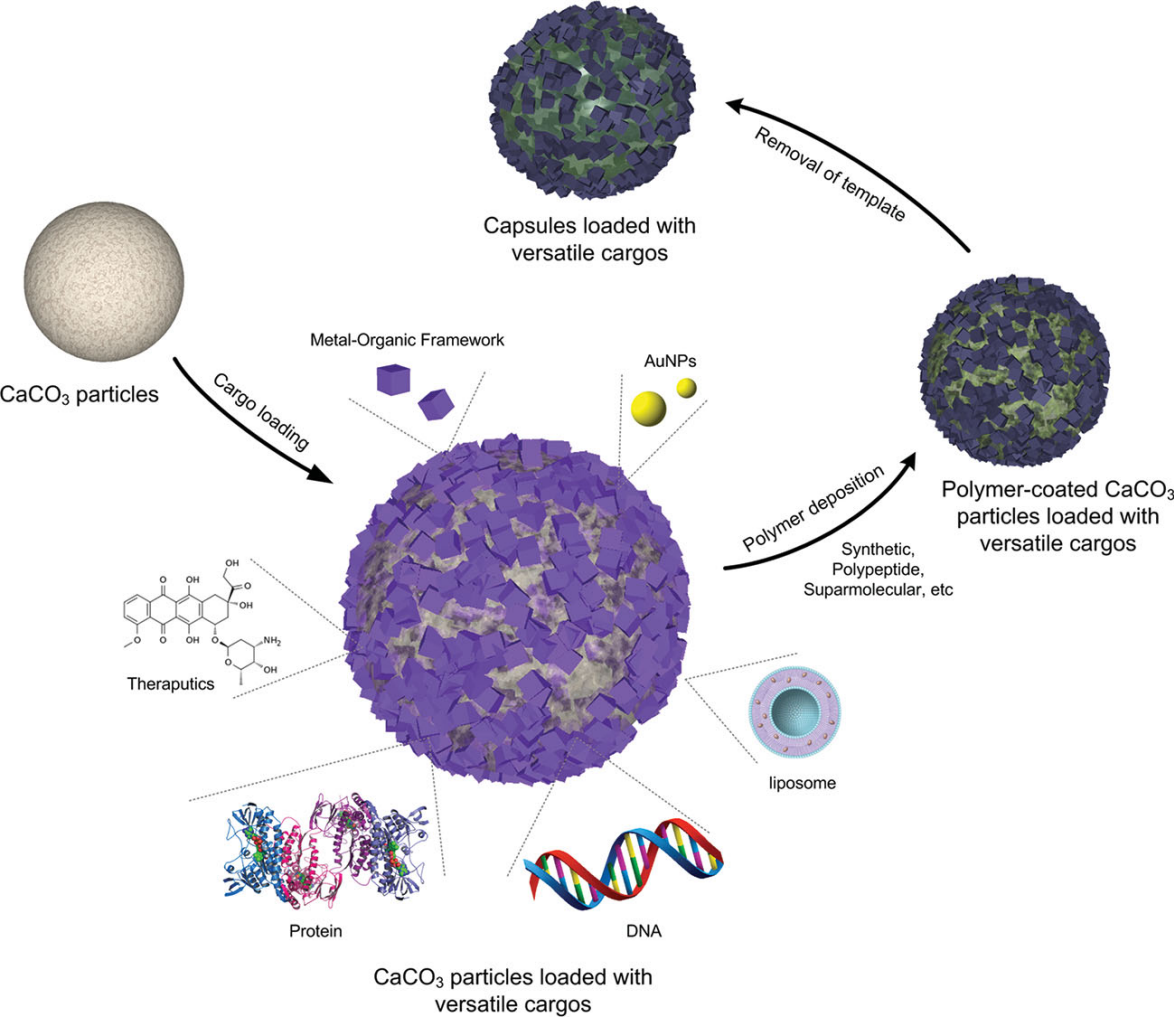
Scheme of the capsule assembly process. Polymer‐stabilized calcium carbonate particles are loaded with diverse cargo, ranging from biomolecules to inorganic particles and then capped with a polymer such as a polypeptide or a supramolecular polymer. After capping, the calcium carbonate core can be removed, yielding highly loaded functional capsules. A detailed table of the cargo, stabilizing polymers, and capping polymers examined in this study can be found in Table S1, Supporting Information.

Herein, we report a method for synthesizing polymer‐stabilized CaCO_3_ particles that are capable of adsorbing a wide variety of cargo (ranging from biomolecules to inorganic and organic materials) for the preparation of a broad range of polymer capsules in under 30 min (in most cases), from template synthesis to core removal (**Scheme** **1**). This technique is not only accomplished in a short time but also in a limited number of steps, which should make it conducive for scale up. Six different types of polymer‐stabilized CaCO_3_ particles (four stabilizing polymers, one of which can be used to produce three distinct sizes) are used to load 15 different types of cargo of various charges and hydrophobicity, comprising sizes from hundreds of nanometers (large metal‐organic frameworks (MOFs)) to below one nanometer (doxorubicin (DOX)) (Table S1, Supporting Information). Polymer capsules could then be prepared after capping with a single polymer layer chosen from seven different capping polymers and removing the CaCO_3_ cores, leading to the formation of pH‐responsive, biodegradable, redox responsive or nonresponsive capsules.

## 2 Results and Discussion

**Figure 1:**
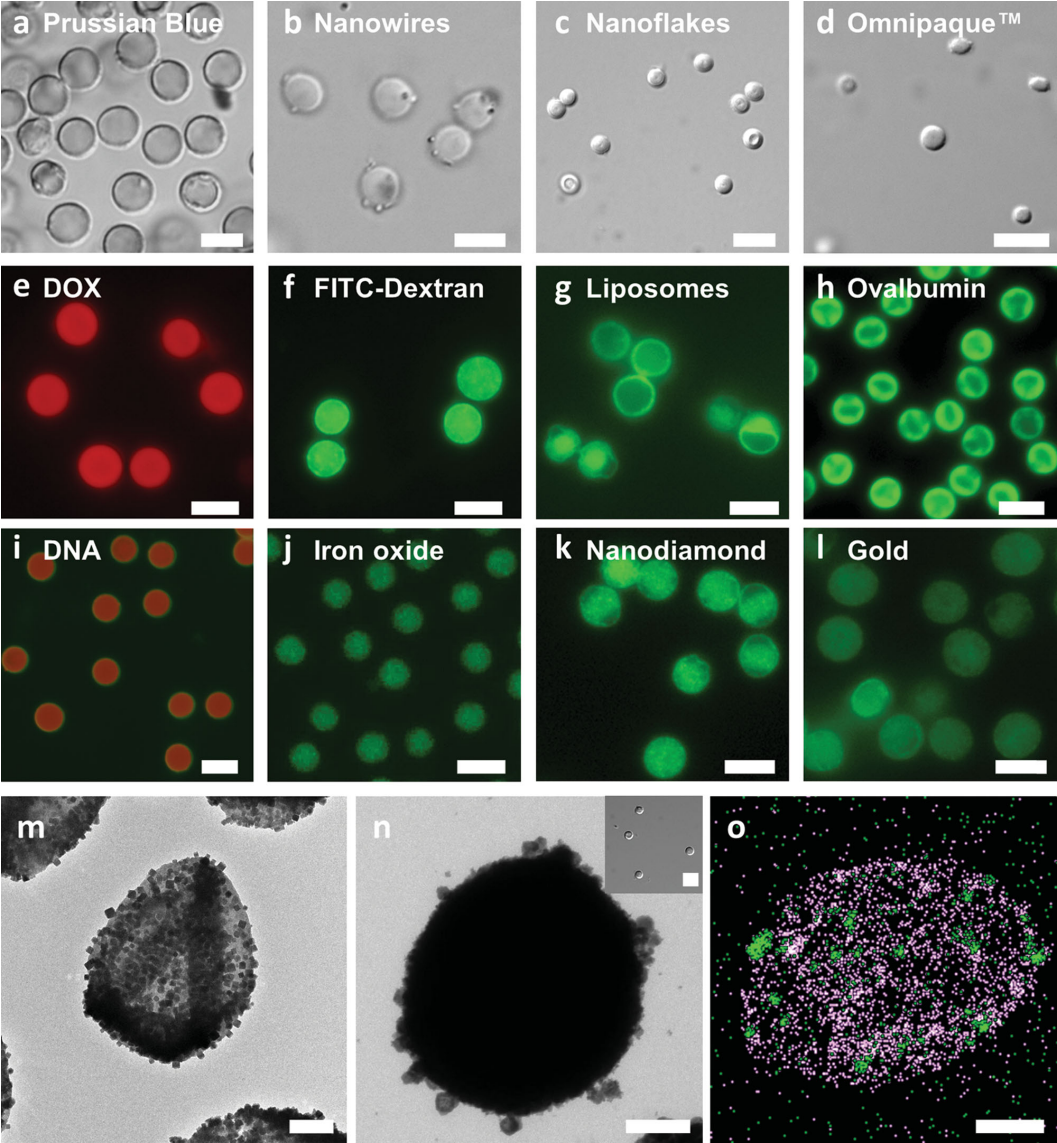
Microscopy images corresponding to capsules loaded with various types of cargo prepared from a–d) and f–o) PSS/PAH and e) PSS/HIS. The fluorescence microscopy images correspond to autofluorescence for e) DOX, j) iron oxide, k) nanodiamonds, and l) gold, and to fluorescent labels for f) fluorescein isothiocyanate (FITC)‐dextran, g) liposomes (FITC‐labeled lipid), h) ovalbumin (FITC), and i) DNA (Alexa Fluor 647‐labeled DNA and FITC‐labeled PAH). m) TEM image of PSS/PAH capsules partially loaded with Prussian Blue cubes. n) PSS/PAH capsule loaded with Prussian Blue cages (inset) with a differential interference contrast (DIC) image of the capsules. o) EDX mapping of a PSS/PAH capsule loaded with Prussian Blue cages (purple corresponds to sulfur from PSS and green corresponds to iron from Prussian Blue cages). The scale bars are a–l) 5 μm and m–o) 1 μm.

As a first example, 4–5 μm PSS–CaCO_3_ particles (Figure S1, Supporting Information) were used to adsorb large MOFs, which are useful for diverse applications such as catalysis,^22^ sensing,^23^ and separations^24^ (**Figure** **1**). These materials were loaded onto the PSS–CaCO_3_ particles in the form of cubes (≈2 wt%; i.e., 1 mg of PSS–CaCO_3_ loaded 0.02 mg of MOF cubes),^25^ wires (≈1 wt%),^26^ flakes (≈14 wt%),^27^ and cages (<1 wt%).^25^ Note that these values translate roughly to mono­layer deposition. A single capping layer of poly(allylamine hydrochloride) (PAH) was then deposited to stabilize the cargo and allowed for the preparation of capsules after CaCO_3_ dissolution with 40 × 10^−3^ m sodium acetate buffer (pH 4) (Figures 1 and S2, Supporting Information). Partially loaded and fully loaded capsules were observed and the presence of iron originating from the MOF cages was confirmed with energy dispersive X‐ray spectroscopy (EDX) (Figure 1). Other inorganic materials, such as gold nanoparticles of different sizes (Figure S3, Supporting Information), capable of catalysis and photothermal reactions,^28^ nanodiamonds (Figure S4, Supporting Information), capable of detecting ionic spin^29^ and useful for biomedical applications,^30^ and fluorescent/magnetic iron oxide nanoparticles^31^ (Figure S5, Supporting Information) were loaded onto the PSS–CaCO_3_ particles and were capped with PAH for the preparation of functional capsules.

The incorporation of small molecules and biomolecules is of interest for biomedical applications and was also investigated. Plasmid DNA (Figure S6, Supporting Information), potentially useful for gene therapy or protein expression,^10^ liposomes (Figure S7, Supporting Information), useful for encapsulating sensitive cargo,^4^, ^5^, ^32^ protein (ovalbumin) (≈7 wt%) (Figure S8, Supporting Information), useful as a model antigen for vaccine delivery,^8^, ^33^ iodine dendrimers (Figure S9, Supporting Information), useful for imaging,^34^ polydextran (Figure S10, Supporting Information), a model cargo,^13^ and DOX (Figure S11, Supporting Information), useful as an anticancer agent,^35^ were all (separately) loaded onto PSS–CaCO_3_ particles and resulted in stable capsules after polymer capping and template removal. The DOX‐loaded capsules (≈33 wt%) were capped with a biodegradable polypeptide, poly(l‐histidine) (HIS) and the release of DOX was investigated in the presence of protease and at different pHs (Figure S11, Supporting Information). Even though HIS is pH sensitive with a p*K*_a_ of roughly 6.0–6.5, the capsules did not leak above or below the p*K*_a_. However, protease allowed for the release of DOX on a rapid time scale, with most of the DOX (>90%) released after 30 min, as monitored by the DOX fluorescence measured in the supernatant. The observed adsorption capacity for varied cargo is most likely due to a combination of factors including capillary forces,^17^, ^20^, ^36^ electrostatic interactions from both the CaCO_3_ itself (positively charged) and from the PSS (negatively charged),^17^, ^19^ and various other interactions such as π–π interactions originating from the PSS. PSS plays a crucial role in the adsorption, as PSS–CaCO_3_ particles have previously been shown to have a high loading capacity for small fluorescent molecules dependent on the presence of the PSS.^37^ Because of these properties the cargo did not leak out of the capsules, even though capsules of similar composition (PAH and PSS) prepared via other techniques are known to be generally permeable to molecules smaller than 200 kDa.^13^

**Figure 2:**
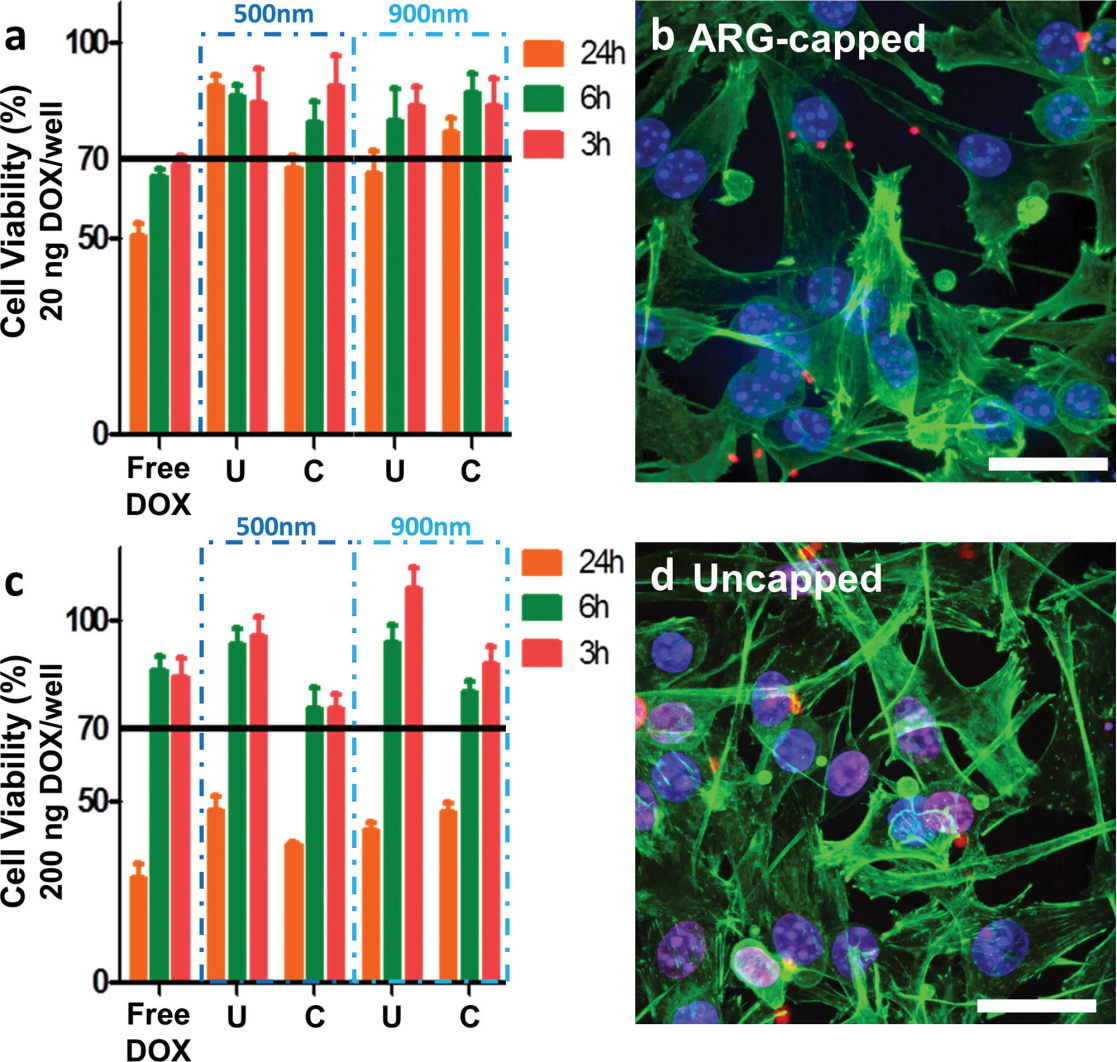
MTT assay cell toxicity results for 500 and 900 nm DOX‐loaded particles and DOX loaded ARG‐capped capsules at different time points for a) 20 ng of DOX per well and c) 200 ng of DOX per well. U – Uncapped DOX–PSS particles (CaCO_3_ removed) and C – capped DOX‐loaded PSS/ARG capsules. Corresponding fluorescence microscopy images of the cells incubated for 6 h with 900 nm; b) uncapped DOX–PSS particles and d) capped DOX‐loaded PSS/ARG capsules (equivalent concentration of 20 ng DOX per well). The cell viability of untreated cells was normalized to 100%. The red corresponds to DOX, the blue corresponds to nuclear staining (Hoechst), and the green corresponds to cell membrane staining (phalloidin‐Alexa Fluor 647). The scale bars are 10 μm.

To further extend this technique, different sizes, shapes, and compositions of polymer‐stabilized CaCO_3_ were obtained. To prepare submicrometer‐sized (900 and 500 nm) PSS–CaCO_3_ particles, Ca(NO_3_)_2_ was used instead of CaCl_2_ and the particles were prepared in less than 20 min rather than overnight.^38^ The 900 nm PSS–CaCO_3_ particles were highly loaded (≈34 wt%) with a pH sensor/fluorophore (naphthofluorescein)^39^ and were capped with PAH to form stable capsules (Figure S12, Supporting Information). The 900 nm and 500 nm PSS–CaCO_3_ particles were loaded with DOX and it was found that they could form stable DOX–PSS particles after template removal without any capping layer (Figure S13, Supporting Information). Cell toxicity was investigated with methylthiazol tetrazolium (MTT) assays at different time points for both sizes of the uncapped DOX–PSS particles and DOX‐loaded PSS/poly(l‐arginine) (ARG) capsules (**Figure** **2**). Corresponding fluorescence microscopy images of the particles and capsules incubated with cells demonstrated that the uncapped DOX–PSS particles broke up quicker than the capped capsules; however, their toxicities were still similar at 24 h. Both the capped and uncapped carriers showed decreased toxicity before 6 h in comparison to free DOX, which together with the cell images suggests that the carriers did not nonspecifically leak DOX before internalization.

**Figure 3:**
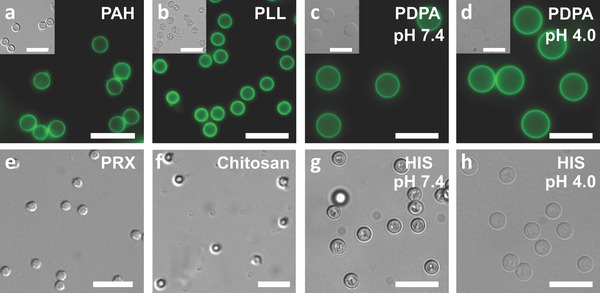
Nonloaded capsules formed with different capping polymers. The fluorescence corresponds to FITC labeling for a) PAH and b) PLL, and to Alexa Fluor 488 labeling for c) and d) PDPA. Both pH‐responsive polymers c) and d) PDPA and g) and h) HIS showed pH‐dependent shrinking (above the p*K*_a_) and swelling (below the p*K*_a_) and became more transparent when hydrated (i.e., below the p*K*_a_). The scale bars are 10 μm and the pH listed on the images corresponds to the pH of the solution when imaging.

Finally, we investigated different polymers for stabilization during particle formation, and also for polymer capping and unloaded capsule formation. Stabilizing polymers, including a polypeptide, poly(l‐glutamic acid) (PGA) (Figure S14, Supporting Information), and natural polysaccharides, dextran‐sulfate (DS) and chondroitin sulfate (CS) were used for particle stabilization during CaCO_3_ particle formation. The DS yielded spherical particles, the PGA yielded primarily nonspherical particles, and the CS yielded a mixture of spherical and nonspherical particles. Pure polypeptide capsules, PGA/poly(l‐lysine) (PLL), could be prepared from the PGA–CaCO_3_ particles. Additionally, the DS–CaCO_3_ particles showed a high loading of DOX (≈20 wt%) and DOX‐loaded DS/ARG capsules demonstrated cytotoxicity against cancer cells in vitro (Figure S15, Supporting Information). Different polymer capping layers were investigated; however, only positively charged polymers could be used as capping polymers because the primary stabilizing force for the capsule shell is electrostatic interactions between the positively charged capping polymer and the negatively charged PSS. For example, neutral poly(ethylene glycol) and negatively charged poly(methacrylic acid) (PMA) could not be used as capping polymers; however, PMA could be loaded into the particles at ≈1 wt%, which is in agreement with the loading of other negatively charged cargo. As mentioned above, the synthetic polymer PAH (≈14 wt%) and biopolymers HIS (≈3 wt%), PLL (≈4 wt%), and ARG were used for preparing nonresponsive or degradable/pH‐sensitive capsules, respectively. Additionally, poly(2‐diisopropylaminoethyl methacrylate) (PDPA) (≈8 wt%), positively charged poly(rotaxane) (PRX),^40^ and chitosan were also used to form capsules (**Figure** **3**). HIS and PDPA yielded pH‐sensitive capsules, while PRX and chitosan produced capsules capable of degrading at elevated pH, as observed by optical microscopy. Additionally, the PRX was prepared with a redox‐sensitive capping group, which also allowed the PSS/PRX capsules to be rapidly degraded after exposure to reducing conditions, as observed by optical microscopy. Only one capping layer was necessary because such a large quantity of polymer is adsorbed by the polymer stabilized CaCO_3_ particles (≈3–14 wt%) in comparison to other film deposition techniques, such as LbL assembly, where significantly less than 1 wt% of polymer is deposited during each layer.^41^ One capping layer was sufficient for capsule formation, as the high surface area of the CaCO_3_ particles and the stabilizing polymer allow for a dense capping layer to be deposited, which leads to denser capsules with less folds than conventional LbL assembly, as observed by transmission electron microscopy (TEM) (Figure S2, Supporting Information).^16^ Unloaded capsules could not be formed without a polymer capping layer (Figure S16, Supporting Information) and the only cargo capable of forming stable particles after core removal without polymer capping was DOX, as demonstrated above.

## 3 Conclusion

Polymer‐stabilized CaCO_3_ particles allow for a comprehensive toolbox to be applied to the preparation of functional, cargo loaded polymer capsules. Using the different sized particles, stabilizing polymers, capping polymers and cargo demonstrated herein, over 500 unique types of loaded and unloaded capsules could potentially be prepared (Table S1, Supporting Information). It is foreseeable that other polymers or thin films, such as metal phenolic network films,^3^, ^42^ could be used for the preparation of the CaCO_3_ particles and for the capping layer, as the examples provided herein are not exhaustive. Similarly, the cargo and therapeutics loaded in this study were chosen as representative classes of model materials highlighting different orders of magnitude in size, different charge, and different hydrophobicity. It is expected that the technique will play a crucial role across scientific studies in numerous fields because no expensive or harsh reagents are required (in most cases) of the preparation steps and because it can take less than 30 min to fully prepare the loaded polymer capsules from the CaCO_3_ particle synthesis to the core removal. Finally, it is noted that because of the highly biocompatible nature of CaCO_3_, the core could be left in depending on the application.

## 4 Experimental Section

*Synthesis of Polymer‐Stabilized CaCO_3_ Particles*: Submicrometer PSS–CaCO_3_ particles and CaCO_3_ particles stabilized with other polymers were obtained by the fast precipitation reaction between Ca(NO_3_)_2_ and Na_2_CO_3_, similar to previous reports.^19^ Solutions (20 × 10^−3^ m) of both Ca(NO_3_)_2_ and Na_2_CO_3_ were separately prepared in the presence of 1 mg mL^−1^ of stabilizing polymer. The stabilizing polymer–Na_2_CO_3_ solution was quickly injected into the stabilizing polymer–Ca(NO_3_)_2_ solution under constant stirring and the reaction was allowed to proceed for 10 min (to form the 500 nm PSS–CaCO_3_ particles, the reaction was allowed to proceed for 5 min instead of 10 min), after which the particles were spun down at 2000 *g* (1 min) and washed three times with ultrapure water. To wash the particles, all but 2 mL of solution was removed from the supernatant at each step, the solution pellet was then mixed vigorously, and finally 20 mL of ultrapure water was added to the tube followed by additional mixing. Larger PSS–CaCO_3_ particles were obtained by adding 2.4 mL of 1 m CaCl_2_ containing 1 mg mL^−1^ PSS into 96.4 mL of ultrapure water, followed by the addition of 1.2 mL of 1 m Na_2_CO_3_ containing 1 mg mL^−1^ PSS. The solution was then stirred for 30 s followed by overnight incubation. These particles were pelleted at 600 *g* (30 s) and washed with ultrapure water three times. The particles were stable when stored at ambient conditions for extended periods of time (upwards of 1 month).

*Cargo Loading*: A 100 μL of a 10 mg mL^−1^ solution of the polymer‐stabilized CaCO_3_ particles was diluted to 300 μL with ultrapure water and 200 μL of 1–10 mg mL^−1^ of cargo dispersed in ultrapure water was added. If the cargo was not readily available in ultrapure water, the pH was adjusted, or in the case of napthofluorescein, dimethyl sulfoxide (DMSO) was used for loading. The solution was mixed vigorously for 1 min before three centrifugation/wash cycles using 2000 *g* were applied for the smaller particles and 600 *g* for the larger particles.

*Polymer Capping and Core Removal*: A 100 μL of a 10 mg mL^−1^ solution of the polymer‐stabilized CaCO_3_ particles was diluted to 300 μL with ultrapure water and 200 μL of 1 mg mL^−1^ polymer solution was added. Note that the polymers were dissolved in pH adjusted ultrapure water, or buffer other than acetate buffers when necessary, with the final pH of the polymer solution 0.5–1.5 pH units below the polymer p*K*_a_. The solution was mixed vigorously for 1 min before three centrifugation/wash cycles were applied. A 200 μL of 40 × 10^−3^ m sodium acetate buffer (pH 4) was then added to the particles, dissolving the templates using the spin speeds mentioned above depending on the template size.

## Supporting information

As a service to our authors and readers, this journal provides supporting information supplied by the authors. Such materials are peer reviewed and may be re‐organized for online delivery, but are not copy‐edited or typeset. Technical support issues arising from supporting information (other than missing files) should be addressed to the authors.

Supplementary
