# Supplementary material for: Versatile Loading of Diverse Cargo into Functional Polymer Capsules
Source: Adv Sci (Weinh). 2015 Jan 29;2(1-2):1400007. doi: 10.1002/advs.201400007 (PMC5115278; doi:10.1002/advs.201400007)
Supplement: Supplementary file 1 — Supplementary [file ADVS-2-0b-s001.pdf]

Copyright WILEY-VCH Verlag GmbH & Co. KGaA, 69469 Weinheim, Germany, 2015.

Supporting Information

for *Adv. Sci.,* DOI: 10.1002/advs.201400007

Versatile Loading of Diverse Cargo into Functional Polymer Capsules

*Joseph J. Richardson, James W. Maina, Hirotaka Ejima, Ming Hu, Junling Guo, Mei Y. Choy, Sylvia T. Gunawan, Lien Lybaert, Christoph E. Hagemeyer, Bruno G. De Geest, and Frank Caruso**

WILEY-VCH

Copyright WILEY-VCH Verlag GmbH & Co. KGaA, 69469 Weinheim, Germany, 2013.

Supporting Information

Versatile Loading of Diverse Cargo into Functional Polymer Capsules

*Joseph J. Richardson, James W. Maina, Hirotaka Ejima, Ming Hu, Junling Guo, Mei Y. Choy, Sylvia T. Gunawan, Lien Lybaert, Christoph E. Hagemeyer, Bruno De Geest, Frank Caruso**

Table S1. List of stabilizing polymer, cargo, and capping polymer used in this study.

Stabilizing Polymers	Cargo	Capping Polymers
Poly(styrene sulfonate) (PSS)<br>Poly(L-glutamic acid) (PGA)<br>Dextran sulfate (DS)<br>Chondroitin sulfate (CS)	Prussian Blue cube (MOF)<br>Prussian Blue cage (MOF)<br>Silver cyanide wire (MOF)<br>Nickel flake (MOF)<br>Plasmid DNA<br>15 nm gold nanoparticles<br>60 nm gold nanoparticles<br>Nanodiamond<br>Iron oxide nanoparticles<br>Liposomes<br>Ovalbumin (OVA)<br>Doxorubicin (DOX)<br>Naphthofluorescein<br>Iodine dendrimer (Omnipaque™)<br>FITC-Dextran	Poly(allylamine) hydrochloride (PAH)<br>Poly(2-diisopropylaminoethyl methacrylate) (PDPA)<br>Poly(L-histidine) (HIS)<br>Poly(L-arginine) (ARG)<br>Poly(L-lysine) (PLL)<br>Chitosan<br>Poly(rotaxane) (PRX)

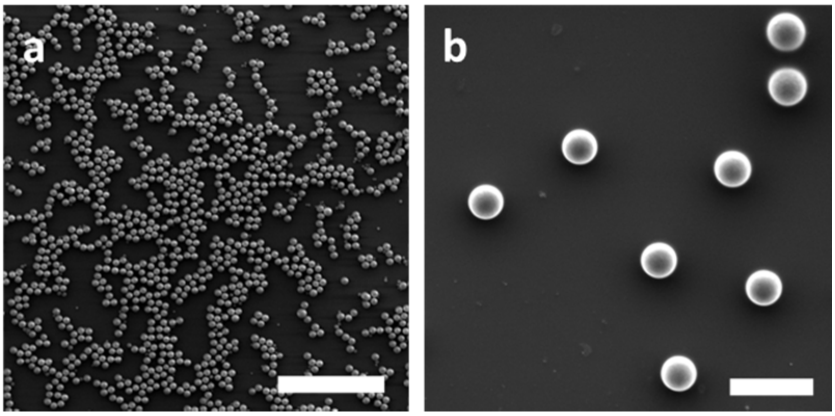

Figure S1. SEM of monodisperse PSS-$CaCO_3$ particles. The scale bars are a) 50 µm, and b) 10 µm.

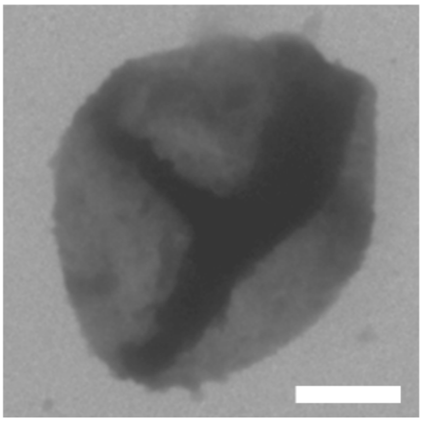

Figure S2. TEM image of a PSS/PAH capsule prepared from PSS-$CaCO_3$ particles. The scale bar is 1 μm.

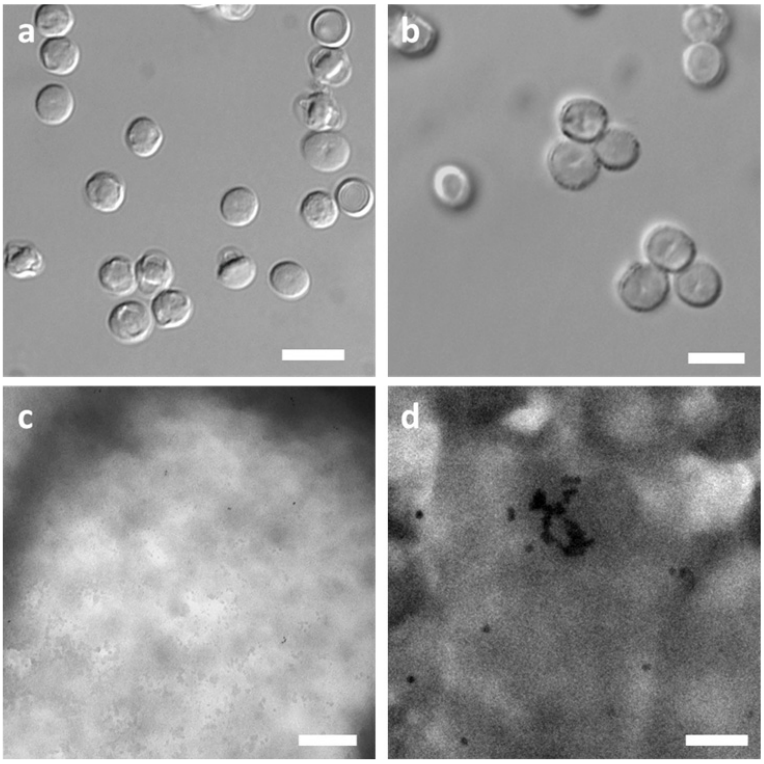

Figure S3. Gold nanoparticle-loaded PSS/PAH capsules. Differential interference contrast (DIC) microscopy images of capsules containing a) 15 nm nanoparticles and b) 60 nm particles. TEM images of capsules containing c) 15 nm nanoparticles and d) 60 nm nanoparticles. The scale bars are a) 10 μm, b) 5 μm, c) 200 nm, and d) 500 nm.

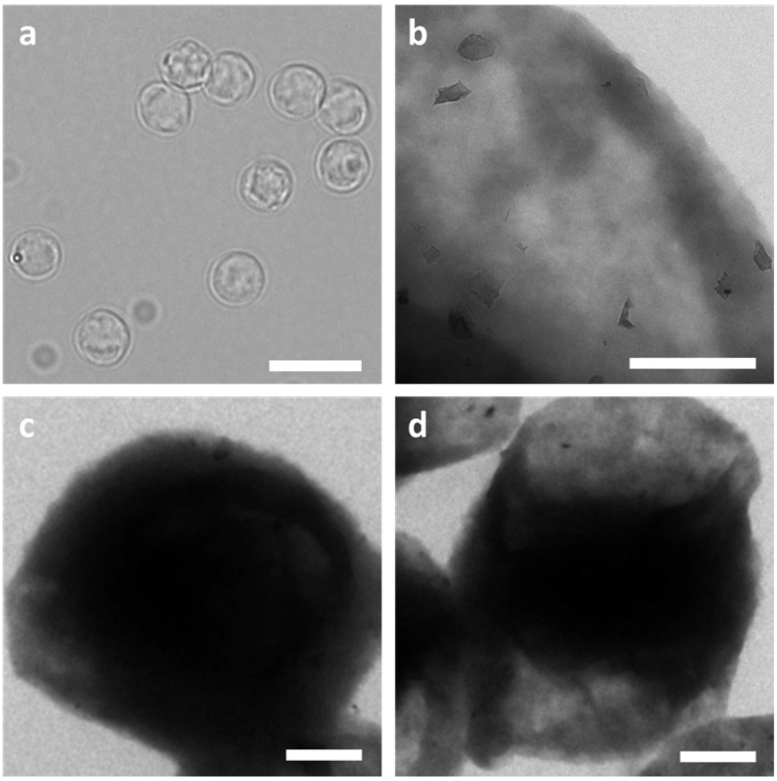

Figure S4. Nanodiamond-loaded PSS/PAH capsules. a) DIC microscopy image of the capsules. TEM images showing, b) the nanodiamonds in the capsule shell, c) capsules fully loaded with nanodiamonds, and d) capsules partially loaded with nanodiamonds where a majority of the nanodiamonds have clustered due to drying. The scale bars are a) 10 µm, b) 500 nm, and c, d) 1 µm.

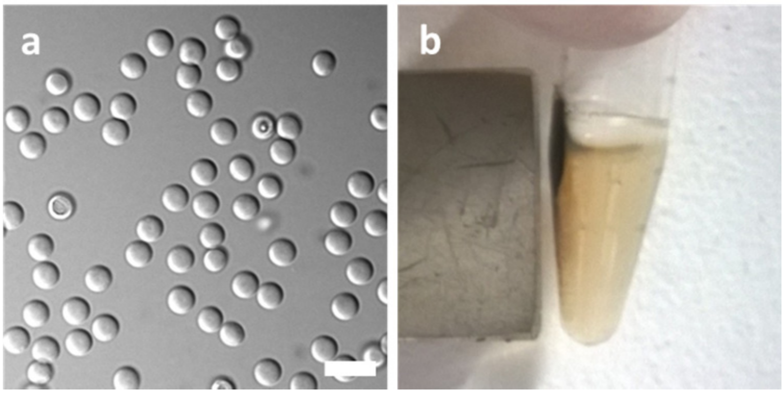

Figure S5. Iron oxide-loaded PSS/PAH capsules. a) DIC microscopy image of the capsules, and b) magnetic response of the capsules. The scale bar is 10 µm.

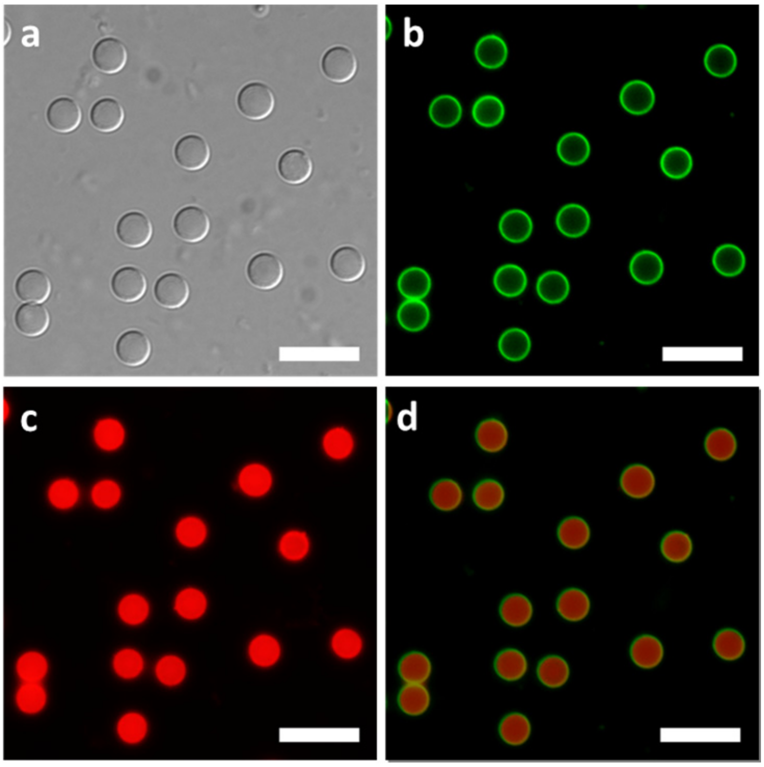

Figure S6. PSS/PAH capsules loaded with fluorescently labeled plasmid DNA. a) DIC microscopy image of capsules. Fluorescence microscopy images corresponding to b) PAH-FITC and c) DNA-Alexa Fluor 647, and d), overlay of fluorescent images (same image is in **Figure 1**). The scale bars are 10 µm.

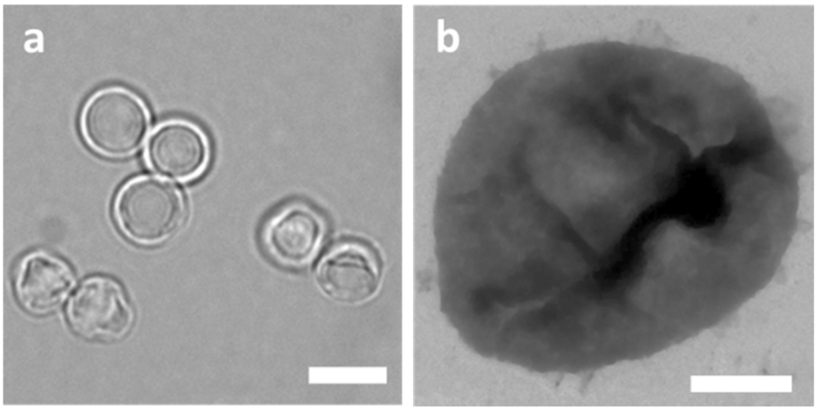

Figure S7. Liposome-loaded PSS/PAH capsules. a) DIC microscopy image of the capsules, and b) TEM image of a capsule. The scale bars are a) 5 µm, and b) 1 µm.

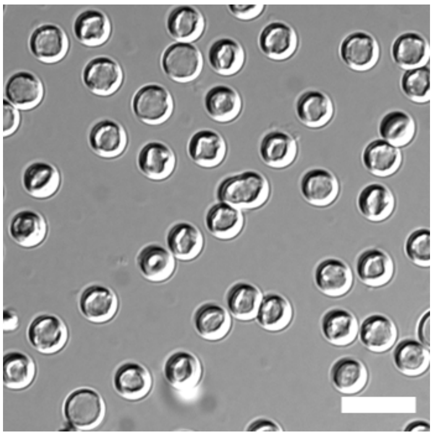

Figure S8. DIC microscopy image of OVA-FITC loaded-PSS/PAH capsules. The scale bar is 10 µm.

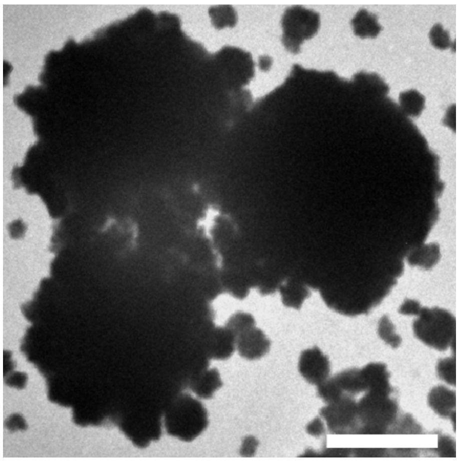

Figure S9. TEM image of iodine dendrimer (Omnipaque™)-loaded PSS/PAH capsules. The scale bar is 1 μm.

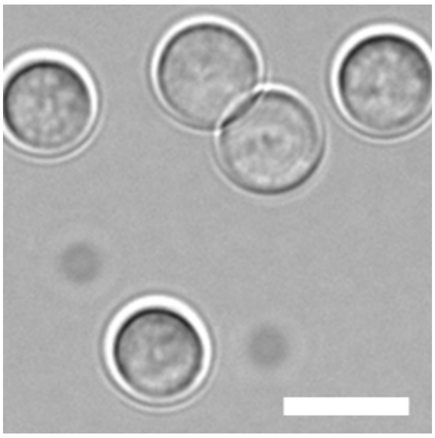

Figure S10. DIC microscopy image of FITC-Dextran-loaded PSS/PAH capsules. The scale bar is 5 μm.

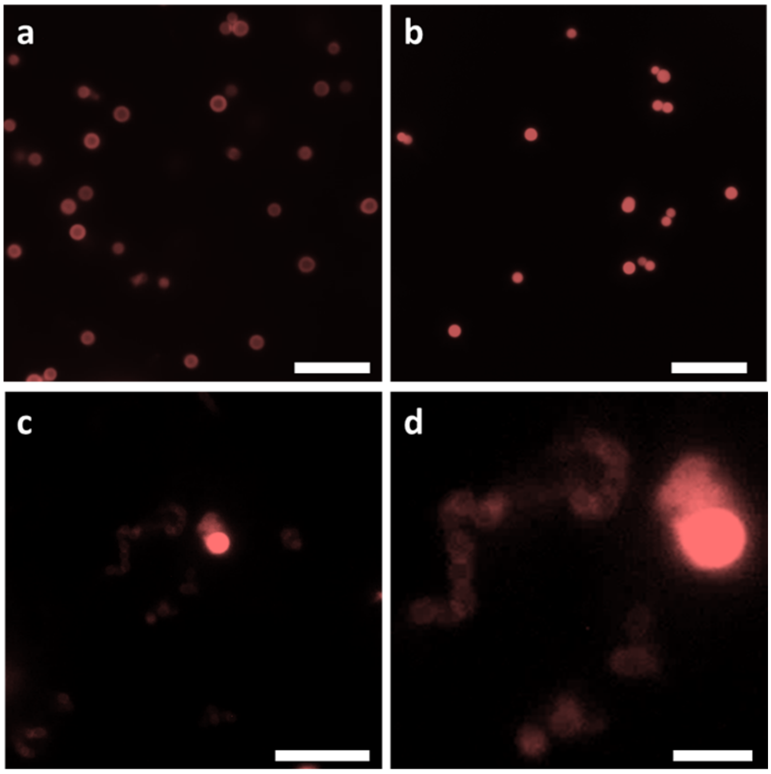

Figure S11. DOX loaded PSS/HIS capsules. Fluorescence microscopy images of capsules incubated for 30 min at a) pH 4, b) pH 7.4, c) pH 7.4 with protease, and d) a magnified image of c) showing shrunken, degrading capsules next to an intact capsule. The scale bars are a-c) 15 µm, and d) 5 µm.

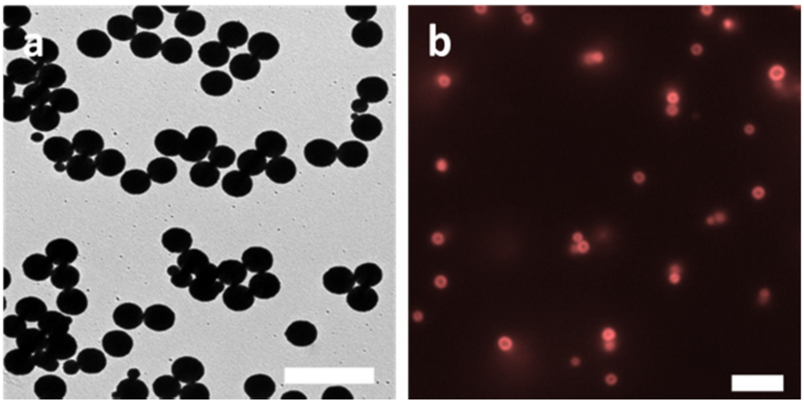

Figure S12. Submicron template particles and naphthofluorescein-loaded PSS/PAH capsules. a) TEM image of 900 nm PSS-$CaCO_3$ particles, and b) fluorescence microscopy image of the 900 nm naphthofluorescein-loaded capsules. The scale bars are a) 2 µm, and b) 5 µm.

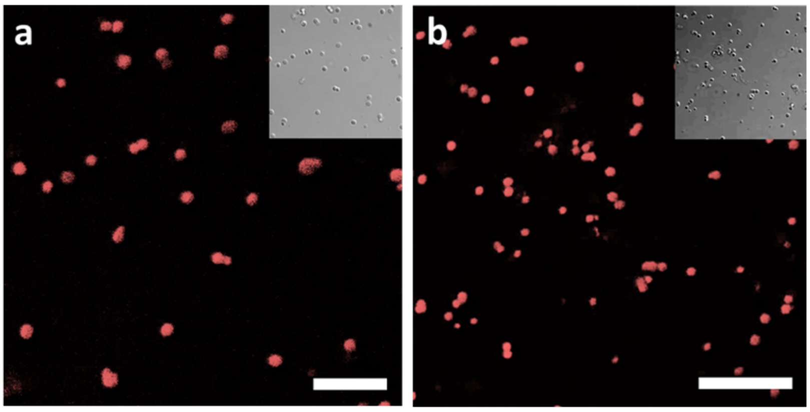

Figure S13. Fluorescence microscopy images inset with DIC images of a) 900 nm DOX-PSS particles without a template and b) 500 nm DOX-PSS particles without a template. The scale bars are 5 µm.

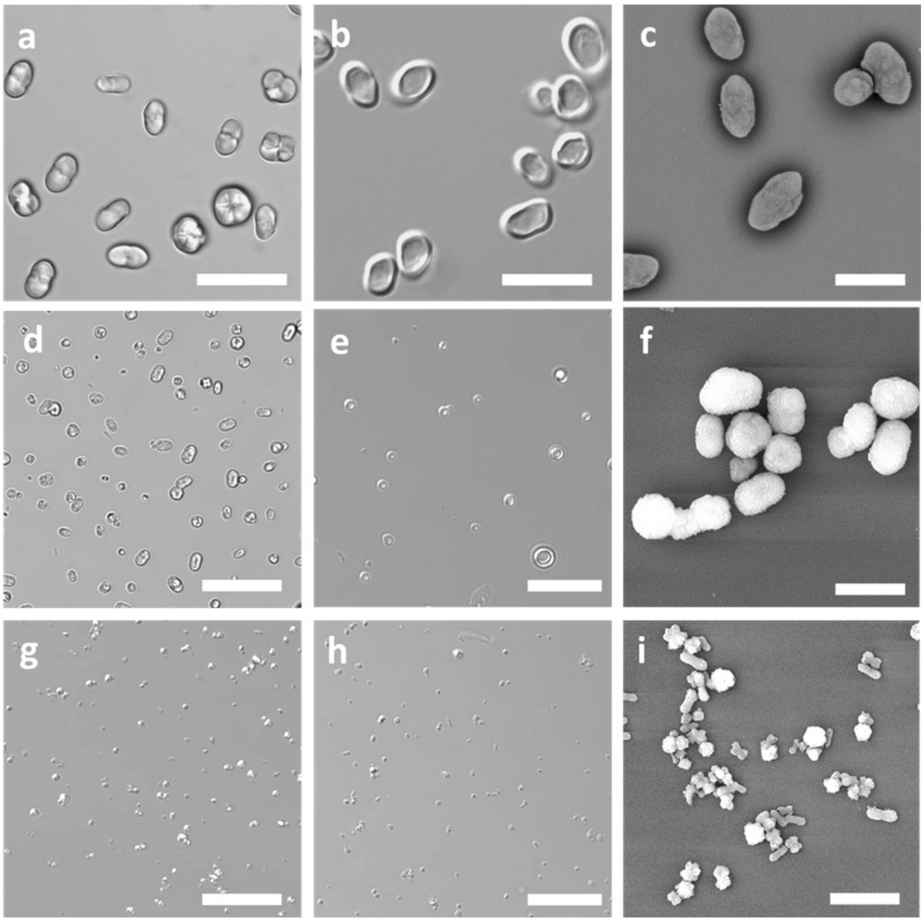

Figure S14. $CaCO_3$ particles with different stabilizing polymers and the corresponding capsules. DIC microscopy images of a) PGA-$CaCO_3$ particles, b) PGA/PLL capsules, d) CS-$CaCO_3$ particles, e) CS/ARG capsules, g) DS-$CaCO_3$ particles, and h) DS/ARG capsules. SEM images of c) PGA-$CaCO_3$ particles, f) CS-$CaCO_3$ particles, and i) DS-$CaCO_3$ particles (note that particles fused upon drying). The scale bars are a, d, e) 20 µm, b, g, h) 10 µm, and c, f, i) 5 µm.

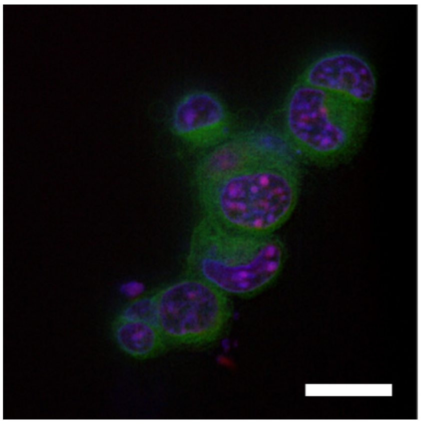

Figure S15. B16 cells incubated overnight with DOX-loaded DS/ARG capsules. Cell clumping and the presence of DOX in the nucleus demonstrate the cytotoxicity. A majority of the cells were lysed and removed with washing. This is a representative image of the remaining cells. The red corresponds to DOX, the blue to nuclear staining (Hoechst), and the green to the cell membrane (CellMask™). The scale bar is 20 µm.

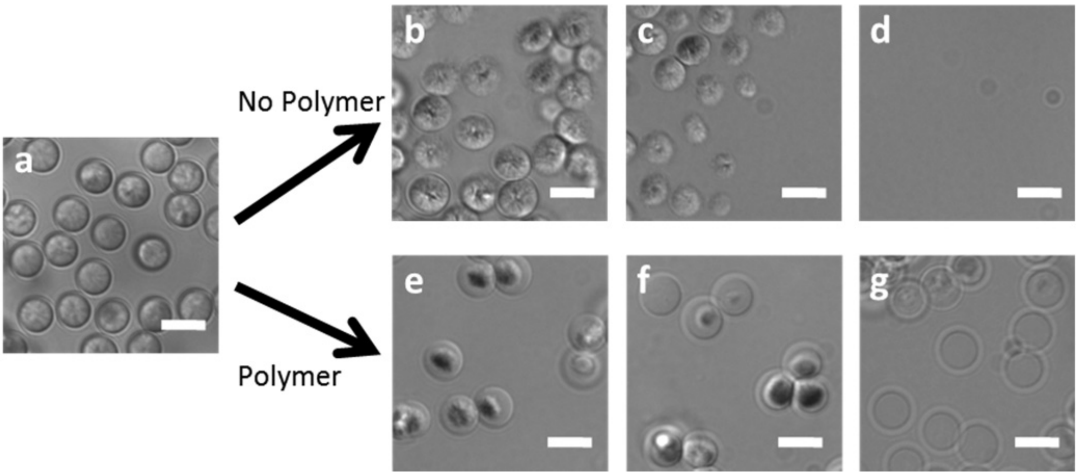

Figure S16. Progression from templates to capsules is dependent on the addition of a capping layer of polymer (PAH in these images). DIC microscopy images of a) the $CaCO_3$ particles before the addition of polymer or acetate buffer, b-d) the dissolution of uncapped particles after the addition of acetate buffer, and e-g) the dissolution of PAH-capped particles after the addition of acetate buffer. The images are taken at different time points, a) 0 s, b, e) 20 s, c, f) 40 s, and d, g) 60 s. The scale bars are 5 µm.
